# Special Issue “Hepatitis B Virus: New Breakthroughs to Conquer an Ancient Disease”

**DOI:** 10.3390/v15112173

**Published:** 2023-10-30

**Authors:** Thomas Tu, Mark W. Douglas

**Affiliations:** 1 Storr Liver Centre, Westmead Institute for Medical Research, University of Sydney and Westmead Hospital, Westmead, NSW 2145, Australia; 2 Sydney Infectious Diseases Institute, University of Sydney and Westmead Hospital, Westmead, NSW 2145, Australia; 3 Centre for Infectious Diseases and Microbiology, Westmead Hospital, Westmead, NSW 2145, Australia

## 1. Introduction

Chronic hepatitis B affects >300 million people worldwide and is a major cause of liver disease, causing ~800,000 deaths each year [1]. While the HBV vaccine can prevent new infections, it is ineffective in people who already have chronic hepatitis B. Modelling based on current practice has predicted that 63 million new infections will occur between 2015 and 2030 [2]. Without a cure, chronic hepatitis B will continue to affect our society until at least 2080 [2]. 

The causative agent in this disease is the hepatitis B virus (HBV). Upon HBV infection, the virus is transported to the nucleus and its genome is converted to covalently closed circular DNA (cccDNA), the template for all viral transcripts. Current therapies block the synthesis of new viral DNA, but existing cccDNA genomes remain untouched. Thus, to cure the infection, cccDNA must be eliminated [3,4].

HBV replicates in almost all liver hepatocytes for decades by evading the immune response. At some point during the infection, the immune system is triggered (through unknown mechanisms) and attacks the infected cells [5]. The resultant inflammation kills the infected hepatocytes, but this response is self-limited (also through unknown mechanisms). The liver eventually settles at a new equilibrium with low levels of virus replication but with a minority of infected cells that continually drives liver injury. This chronic inflammation can lead to cirrhosis and liver cancer and is responsible for the 100-fold increased risk of liver cancer associated with HBV. 

People with chronic hepatitis B can receive life-long therapy that can reduce (but not eliminate [6]) the risk of liver cancer; however, there is no cure for chronic hepatitis B, fueling stigma and discrimination [7]. Given liver cancer’s dismal 5-year survival (~20%), the greatest health impact will come from preventing it by removing the underlying viral infection. 

In this Special Issue, we have brought together a broad range of research on the hepatitis B virus (HBV) to enhance the sector’s understanding of the disease and provide additional avenues for its elimination as a public health threat. A total of 15 manuscripts were included, all highlighting the numerous barriers still to be overcome to address the effects of HBV-associated disease and its psychosocial impacts. 

## 2. Fundamental Understanding of HBV Biology, Transmission, and Replication

Several studies focused on the underlying molecular viral factors that govern HBV transmission and replication. Stone et al. investigated the biological features of the genomes of domestic cat hepatitis virus, a recently discovered relative of HBV. They found that while the domestic cat hepatitis (DCH) virus was not prevalent in the blood and liver of cat populations in the USA, the single isolate they did find had mutations consistent with double-stranded linear DNA formation. This suggests that DCH replicates similarly to HBV and could in future be used as a model for human HBV replication and pathogenesis.

Nakanishi et al. found a new replication factor for HBV, the nuclear importin KPNA2. They found that HBV entry through this pathway could be partially inhibited (by 1.5 to 3-fold) using ivermectin at micromolar concentrations in both hepatoma and primary hepatocytes. While this is unlikely to be a treatment by itself, it has revealed a novel understanding of HBV nuclear import and could inform factors restricting HBV infection in animal models. 

Factors controlling HBV transcriptional activity were investigated by both Doan et al. and Song et al. Doan et al. focused on cellular factors and their interactions with nuclear HBV DNA using super-resolution microscopy, single-molecule fluorescence in situ hybridisation, and chromatin pull-down assays. As part of their key results, they found that the cellular protein DOCK11 appears to be colocalised to, complexed with, and controlled the transcriptional activity of HBV cccDNA. 

Song et al., on the other hand, focused on viral factors, finding that a deletion in the HBx open reading frame (aa128-133) reduces HBV replication. Indeed, this mechanism provided an explanation for the reduced observed rate of mother-to-child transmission in mothers with circulating HBV DNA genomes with these mutations in their clinical cohort. Sirilert et al. investigated similar phenomena, looking at genomic sequences of HBV detected in the cord blood and placenta of pregnant women. They found no consistent viral mutations that were significantly associated with detection in these organs, suggesting that mother-to-child transmission may be a highly multifactorial phenomenon. 

## 3. HBV-Associated Pathogenesis

Viral proteins were investigated as drivers of liver disease by multiple groups in this Special Issue. Han et al. characterised a potential pathway of cancer induction by the HBx protein through ERK, CREB, and p90 ribosomal S6 kinase 2. Padarath et al. provided an overview on the potential pro-carcinogenic roles of HBeAg, a viral protein with reported immunomodulatory functions. Finally, Zhang et al. posited a hypothesis that capsid–antibody complexes (formed by the secretion of naked capsids into circulation where they are immediately complexed by anti-HBc antibodies) could drive intrahepatic inflammation. These papers describe the multifactorial, complex, and subtle ways that HBV may trigger cellular pathways to drive inflammation and cancer. 

Meanwhile, a clinical study by Makuza et al. showed that concurrent HBV infection in all cases of other liver diseases (e.g., fatty liver or hepatitis C virus infection) was linked to significantly worse liver-related mortality. While further research is needed into the pathogenic mechanisms underlying the interactions between HBV and these various conditions, this report suggests that they are synergistic (at least with fatty liver disease).

Finally, Miodownik et al. showed that some comorbidities can lead to changes in observed pathogenesis. When comparing transient elastography and two-dimensional shear wave elastography as measures of liver fibrosis, they found that diabetes mellitus can lead to an overestimation of fibrosis level by transient elastography. This should be kept in mind as a potential confounder for clinical studies. 

## 4. HBV Cure

Mitigation of HBV-associated disease could come through new therapies that induce a functional cure, i.e., loss of circulating HBsAg. Coffin et al. investigated the natural history of clinical cohorts to identify those patients most likely to seroconvert. They found that a low HBsAg level was associated with a significantly greater chance of HBsAg-clearance within 1 year, consistent with previous studies suggesting that these patients should be a priority population for the testing of new curative therapies. 

One of the most promising approaches for inducing a functional cure are nucleic acid polymers. Vaillant provided an overview of the proposed mechanisms and explanations for the vast difference in their observed effect compared to other nucleic acid-based therapies (e.g., antisense oligonucleotides and short interfering RNA).

## 5. Implementation of Optimal HBV Care

Even if a cure were to be discovered tomorrow, our health system is currently insufficient to provide optimal care (even with today’s relatively cheap diagnostics and treatments). Implementing new treatments at scale to achieve a cure would take years to achieve, particularly for those who have the greatest need. Wallace et al. interviewed stakeholders in the sector and found potential solutions that could be implemented now to prepare for such a discovery, including changing health policies; developing resourcing options; and ensuring that health service delivery models are appropriate for the affected community. 

Broader coverage of vaccination must be implemented to control HBV. Kazmi et al. found in a Pakistani University staff and student population that only 0.3% of people reported being vaccinated against HBV. They also found that although HBV promotion programs increased knowledge about the condition, whether this translates into action and long-lasting awareness remains to be seen. 

Another method to expand care is to simplify the criteria for treatment. Geeratragool et al. developed a clinical algorithm for determining whether to provide treatment to a given patient according to EASL guidelines, based only on HBeAg, platelet count, alanine transaminase, and albumin tests, rather than the more expensive, less widely available HBV DNA PCR test. Validation and implementation of this algorithm could easily expand treatment to those who would most benefit from it, particularly in remote or resource-limited settings. 

## 6. Concluding Remarks

This Special Issue has touched on all aspects of hepatitis B, from the fundamental virology surrounding its replication all the way to implementation of appropriate health care in the affected community. All these issues need to be addressed through a multipronged, multidisciplinary approach to most effectively ensure that hepatitis B is eliminated as a public health threat worldwide.

## Data Availability

Not applicable.
